# Evaluation of the prognostic role of SOX2 as a tumor stem cell marker in odontogenic cysts and tumors: clinical, radiographic and immunohistochemical correlation

**DOI:** 10.1186/s12903-025-07065-0

**Published:** 2025-11-01

**Authors:** Haneen Mahmoud Zeen El-Abdeen, Mohamed Abdel-Monem Tawfik, Sherif Yousef Elnagdy, Noha Ahmed Mansour

**Affiliations:** 1 Department of Oral and Maxillofacial Surgery, Faculty of Dentistry, Mansoura University, Mansoura, Egypt; 2 Oral Pathology Department, Faculty of Dentistry, Mansoura University, Mansoura, Egypt

**Keywords:** SOX2, Odontogenic keratocyst, Ameloblastoma, Dentigerous cyst; immunohistochemistry

## Abstract

**Background:**

Odontogenic cysts and tumors are highly encountered jaw lesions with varying clinical characteristics and disease behavior. Its pathogenesis may involve the existence of tumor stem cells. SOX2 transcription factor is expressed in embryonic and adult stem cells and exerts a potent influence on maintaining pluripotency. SOX2 expressing cells would account for the aggressive nature and recurrence of some odontogenic pathologies. To evaluate the prognostic role of SOX2 stem cell marker in odontogenic cysts and tumors and determine whether its expression is associated with the clinico-biological behavior of these lesions, this study aimed to assess the immunoexpression of the stem cell marker SOX2 in ameloblastoma, odontogenic keratocyst and dentigerous cyst and correlate SOX2 immunohistochemical staining scores to clinical and radiographic findings and recurrence of these lesions.

**Methods:**

Forty-Five surgical specimens were included in this study, 15 ameloblastomas (Ab), 15 odontogenic keratocysts (OKC) and 15 dentigerous cysts (DC). An immunohistochemical (IHC) study using the SOX2 Rabbit Polyclonal Antibody was done to evaluate SOX2 expression. A semi-quantitative method for subjectively scoring percentage and intensity of SOX2 staining was carried out. Statistical correlation to clinical data, radiological findings and recurrence were analyzed.

**Results:**

Nuclear SOX2 expression was strong positive within OKC specimens in both basal and sub-basal layers, followed by Ameloblastoma which showed nuclear and cytoplasmic reaction, while most cases of DC recorded low positive scores. Significant difference in SOX2 (IHC) staining scores was found between (OKC) and (DC) (P = < 0.001*), as well as between (Ab) and (DC) (*P* = 0.012*), (OKC) showed a significant higher SOX2 expression than ameloblastoma (*P* = 0.048*). A significant positive correlation was found between SOX2 expression and both cortical bone perforation and recurrence (*P* = 0.040*, 0.001*), while no significant correlation to age, gender, root resorption or radiographic loculation (*P* = 0.874, 0.162, 0.062, 0.137).

**Conclusion:**

SOX2 is a reliable marker for tumor stem cells (TSCs) within benign odontogenic lesions. Positive correlation of SOX2 expression to cortical bone perforation and recurrence of these lesions point to the aggressive biological behavior, clinical outcome and poor prognosis. The prognostic role of SOX2 is of great value to improve treatment of odontogenic lesions.

**Trial registration:**

>This study was registered in ClinicalTrials.gov PRS (https://register.clinicaltrials.gov/) under identification number NCT06833840 on 01/21/2025.

## Background

Odontogenic cysts and tumors are among the most often observed pathological lesions in the head and neck region, which can be defined as clinico-pathological entities with inherent degrees of biological aggressiveness and wide range of disease behavior [1]. Considerable variation in the clinicopathologic features can sometimes be challenging and increase the chance of misdiagnosis. The prognosis of these lesions varies depending on their biological behavior, recurrence potential and treatment approach; some lesions have excellent outcomes when diagnosed early, while others are aggressive and require long term follow up [2]. 

Ameloblastoma (Ab) is the most common odontogenic epithelial tumor, accounting for 9% to 11% of odontogenic tumors [3]. It is represented by slow growth, asymptomatic swelling and/or perforation of the cortical bone and might grow into massive proportions causing facial deformity [4]. Although Ab is a benign odontogenic tumor of jawbones, it is characterized by its aggressive local invasion and high recurrence incidence up to 55%−90%, which necessitate precise histological diagnosis and radical surgical treatment. Additionally, few reports of distant metastatic ameloblastomas were documented [5, 6]. The high level of tumor stem cells in Ab may represent a cause for tumor recurrence [7]. 

Odontogenic Keratocyst (OKC) is a distinctive type of odontogenic cysts with an aggressive clinical potential and high recurrence rate (25–60%) [8]. It differs from typical cysts in growth pattern and biological behavior, its growth is triggered by the active proliferation of the epithelial lining rather than the osmotic pressure of the cystic fluid. As OKC grows through extension rather than expansion, it develops antero-posteriorly with limited initial indication of cortical expansion. The destructive nature of OKC and recurrence potential has many theories for the cyst to be consider a tumor [9]. 

Another common cyst of non-inflammatory odontogenic origin, is the dentigerous cyst that arises from the pericoronal tissue of an impacted tooth [10]. Clinically, DCs are often asymptomatic and could be diagnosed incidentally during an oral check-up, but may occasionally cause enormous swelling and dental displacement. Radiographically, they may resemble an OKC or an Ab. DC has good prognosis and rarely recurs, however radiolucencies greater than 4 mm are thought to indicate more aggressive behavior, which can result in tooth displacement [11]. 

A set of genetic and molecular alterations are involved in the pathogenesis, progression and clinical behavior of odontogenic lesions. The exact mechanism remains unclear, recent studies suggest that it may be evolved by a stem cell population called the tumor stem cells (TSCs) [12]. 

SOX2 is a characteristic member of the SOX family of transcription factors, which is considered a subpopulation of stemness-related genes. It is essential for early development, maintenance and self-renewal of undifferentiated stem cells. It is evident in embryonic and adult stem cells, and play an essential role in maintaining pluripotency [13]. It has been found to be related to oncogenic signaling pathways, controlling tumor cells by affecting their fate, proliferation and apoptosis [14]. 

SOX2 is a principle marker for dental epithelial and mesenchymal stem cells associated with tooth renewal and replacement [15]. Recent studies indicate that SOX2 can be a potential novel biomarker to distinguish between malignant, and benign lesions as it is a specific and sensitive marker for high-grade lesions. Furthermore, it has been linked to the process of tumor initiation, destructive clinical course and poor prognosis [16]. 

Variable SOX2 expression was noted in different types of benign and malignant neoplasms [15, 17, 18]. This has been linked to the stem-like characteristics of tumor cells. Poor patient prognosis was associated with elevated SOX2 expression in cancers of the oral cavity, esophagus, breast, liver, rectal and prostate [19]. Available data in literature suggest that SOX2 is expressed differently in odontogenic cysts and tumors which may reflect their different histogenesis, however, few research correlate SOX2 expression to clinical and radiological findings in benign odontogenic cysts and tumors. Predicting biological behavior and prognosis of these common lesions will greatly aid in patient management and improve treatment outcomes.

In order to evaluate the prognostic role of the stem cell marker SOX2, and determine whether its expression in Ab, OKC, and DC is associated with the clinico-biological behavior of these lesions, this study aimed to assess the immunoexpression of SOX2 in these tissues and correlate SOX2 immunohistochemical staining score to clinical data, radiographic findings and recurrence ratio.

## Methods

### Patient selection

Forty-five patients diagnosed with benign odontogenic cysts or tumors (Ab, OKC and DC) either in maxilla or mandible and needed surgical treatment were selected from the Out-Patient Clinic of Oral and Maxillofacial Surgery Department, Faculty of Dentistry, Mansoura University. No age or gender preference was specified in this study. Histopathological examination and immunohistochemistry (IHC) staining of tissue samples were done at Oral Pathology Department Faculty of Dentistry, Mansoura University.

### Ethics and consent

All patients were informed about the aim of the designed study, complete treatment plan and a written consent was obtained. The institutional Review Board (IRB) of the Faculty of Dentistry, Mansoura University, Mansoura, Egypt, approved the current study in compliance with the seventh revision of the Helsinki Declaration in 2013 under protocol number (No. A06010222). It is registered in Clinical-Trials.gov PRS (https://register.clinicaltrials.gov) under identification number NCT06833840 on 01/21/2025.

### Sample size calculation

Sample size calculation was based on mean of SOX2% area among ameloblastoma and odontogenic keratocyst obtained from previous research [20]. Using G*power version 3.1.9.4 [21] to calculate sample size based on effect size of 1.9333975, 2-tailed test, α error = 0.05 and power = 95.0% then a sample of 15 cases per group would be required.

### Study design

This is a prospective immunohistochemical study through which fifty-five patients with suspected odontogenic cysts or tumors underwent further clinical and radiographic assessment. Incisional or excisional biopsy was carried out according to the lesion size and site, histopathological diagnosis of all tissue specimens to confirm lesion type was done based on 2022 WHO Classification of Head and Neck cysts and tumors [22]. 

After exclusion of non-eligible lesions, forty-five specimens were included in this study and were divided into 3 groups:

- Group A: including 15 cases of Ameloblastoma (Ab).
- Group B: including 15 cases of Odontogenic Keratocyst (OKC).
- Group C: including 15 cases of Dentigerous Cyst (DC).

### Surgical procedures

After detailed data collection, a complete clinical and radiographic examination (Panoramic x-ray and Cone Beam Computed Tomography) was done for each patient. Lesion site, size, pattern of radiolucency, relation to anatomical landmarks and buccal or lingual cortical bone perforation were assessed.

An incisional or excisional biopsy was obtained according to the lesion size, the tissue specimens were fixed in 10% neutral formalin and were sent to the Oral Pathology Department, Faculty of Dentistry, Mansoura University where it was preserved into paraffin blocks and histopathologically diagnosed using hematoxylin and eosin staining. According to the diagnosis of each lesion, suitable surgical intervention including marsupialization followed by enucleation, enucleation and curettage, marginal or segmental resection were utilized in the management of different cases in this study.

### Decalcification process

Specimens that required decalcification (i.e., those containing bony fragments) were subjected to gentle EDTA-based decalcification (10% ethylenediaminetetra-acetic acid). This method was specifically chosen because EDTA preserves antigenicity much better than strong acid-based decalcification, thereby minimizing the risk of significant degradation of tissue antigens. The duration of decalcification was also carefully monitored and limited to the minimum necessary to soften the tissues to be sectioned, ensuring reliable immunohistochemical results.

### Immunohistochemical marker

Rabbit polyclonal antibody for Sex-determining region Y (SRY)-box 2(SOX2), BIOCYC Gesellschaft für Biotechnology, Germany (Catalog No. 2-SO108-13, 7 ml Ready-to-use).

### Immunohistochemical staining [23]

Sections of 4 µ thickness were cut from paraffin blocks, mounted on negatively charged Opti plus slides for assessment of SOX2 using a universal kit (1.0 Poly HRP DAB Kit). After being deparaffinized in xylene, the sections were rehydrated in alcohol at decreasing concentrations. Antigen Retrieval was performed. After 10 min of immersion in citrate buffer PH6, the slides were heated, blocked for 30 min using 1.5% horse serum from “Santa Cruz Biotechnology,” and then diluted in phosphate buffered solution (PBS).

Incubation of primary antibodies of rabbit polyclonal anti-SOX2 at room temperature (45 min). Slides were washed with PBS (3 min). The slides were then incubated with the secondary antibody for 25 min at room temperature then were washed in PBS for 3 min. Slides were treated with streptavidin–biotin enzyme reagent “DAKO, Denmark” (10 min) and rewashed in PBS (3 min).

To create color, drops of “3.3 Diaminobenzidine tetrahydrochloride” (DAB) were used as a chromogen. Slides were incubated (10 min) and then washed in PBS for 3 min. DAB incubation time was carefully standardized and monitored. It was consistently applied across all samples to ensure comparability of staining intensity [20, 23]. 

Mayers hematoxylin was used as a counterstain, and sections were fixed for three minutes using a mounting media based on xylene. Positive immune deposits were detected as brown spots. As positive control, Squamous Cell Carcinoma (SCC) was used for confirmation of the marker expression as it is a well-established epithelial malignancy known to exhibit strong and consistent Sox2 expression, thereby providing a reliable and reproducible benchmark for antibody reactivity [24, 25]. Negative controls were prepared using non-immune serum.

### Evaluation of immunohistochemical staining

####  Semi-quantitative method for subjectively scoring the immunohistochemical staining

Immunohistochemically stained sections were interpreted independently by two pathologists. The immune reactivity for SOX2 was assessed by a scoring system based on the both percentage and intensity of staining [26]. Specimens were considered to be positive for staining when the cells had a brown nucleus.

Immune reactivity was evaluated by scanning the slides under a light microscope at 200x magnification. Five fields were selected that were rich in lesional cells. The percentage of staining was graded from (0–4), 0 = negative, 1 = 1:25%, 2 = 26:50%, 3 = 51:75, and 4 = 76:100%. The intensity was interpreted from (0–3) as 0 = negative, 1 = weak, 2 = moderate and 3 = strong.

The percentage and intensity scores were then added to obtain a total score, which ranged from 0 to 7. The cases were categorized into one of the following categories according to their overall scores: Negative expression = 0 points, Low expression = 1–3 points and High expression = 4–7 points.

### Clinical and radiological correlation

Statistical correlations of SOX2 immunohistochemical total staining scores to clinical data including patient age, sex and recurrence, and to radiological findings including lesion site, radiographic loculation pattern, root resorption and buccal or lingual cortical bone perforation were analyzed.

### Statistical analysis

Data was fed to the computer and analyzed using GraphPad Prism 8 (GraphPad Software). Normally distributed data were presented as mean, and standard deviation (SD) values, the One-way ANOVA followed by post hoc Tukey’s multiple comparison test were used to compare between them. While non-normally distributed data were presented as median, and range values, the Kruskal-Wallis test followed by post hoc Dunn’s multiple comparisons test were used to compare between these groups. Chi-square tests of goodness of fit and independence were used to compare the categorical data groups. Spearman’s rank correlation coefficient was used where the value *r* = − 1 means a perfect negative correlation and the value *r* = 1 means a perfect positive one. The significance of the results obtained was judged at the (0.05) level.

## Results

### Clinical results

As shown in (Table 1); The mean patients age of Ab, OKC and DC groups were 33.80 ± 14.65, 32.67 ± 15.01, and 28.07 ± 10.87 years respectively with no statistically significant difference (*P* = 0.48). Twenty-seven patients (60%) were males while eighteen patients (40%) were females. The number of males and females in each group was provided in Table 1, along with the corresponding percentages. Thirty-eight lesions were found in mandible with posterior mandible predominance (6 in anterior mandible and 32 in posterior mandible) while seven lesions were found in maxilla (2 in anterior maxilla and 5 in posterior maxilla). The distribution of number and percentage of lesions sites between groups were shown in (Table 1). The results showed no statistically significant difference in the maxillary and mandibular distributions among studied groups (*p* = 0.31). Five patients included in this study were received as recurrent cases, distributed as 3 (20%), 2 (13.3%), 0 (0%) among Ab, OKC and DC groups respectively. The histopathological assessment of recurrent cases of Ab revealed that 2 cases were conventional Ab of follicular histopathological variants, and the third case was unicystic Ab with mural histopathological variants.

**Table 1:** Clinical data (patients age, sex, lesion site, patients received as recurrent)

	Sex		Age	Site		Patients Received as recurrent
	Male *n* (%)	Female *n* (%)	Mean ± SD	Maxilla Mandible		
				Ant post *n* (%) *n* (%)	Ant Post *n* (%) *n* (%)	
Ab	10 5 (66.7%) (33.3%)		33.80 ± 14.65	1 0 (6.6%)	4 10 (26.7%) (66.7%)	3 (20%)
OKC	9 6 (60%) (40%)		32.67 ± 15.01	1 2 (6.6%) (13.3%)	0 12 (80%)	2 (13.3%)
DC	8 7 (53.3) (46.7)		28.07 ± 10.87	0 3 (20%)	2 10 (13.3%) (66.7%)	0
Test P value	X^2^ = 0.556 *P* = 0.46		F = 0.743 *P* = 0.48	X^2^ = 7.113 *P* = 0.31		X^2^ = 3.15 *P* = 0.07

*Ab* Ameloblastoma, *OK* Odontogenic keratocyst, *DC* Dentigerous cyst, *SD* Standard deviation, *Ant* Anterior, Post: Posterior, *n* number of patients, *X*^2^ Chi-square test, *F* One way ANOVA

P: not significant (at level *p* < 0.05)

### Radiographic results

#### Radiographic locular pattern

**Fig. 1:**
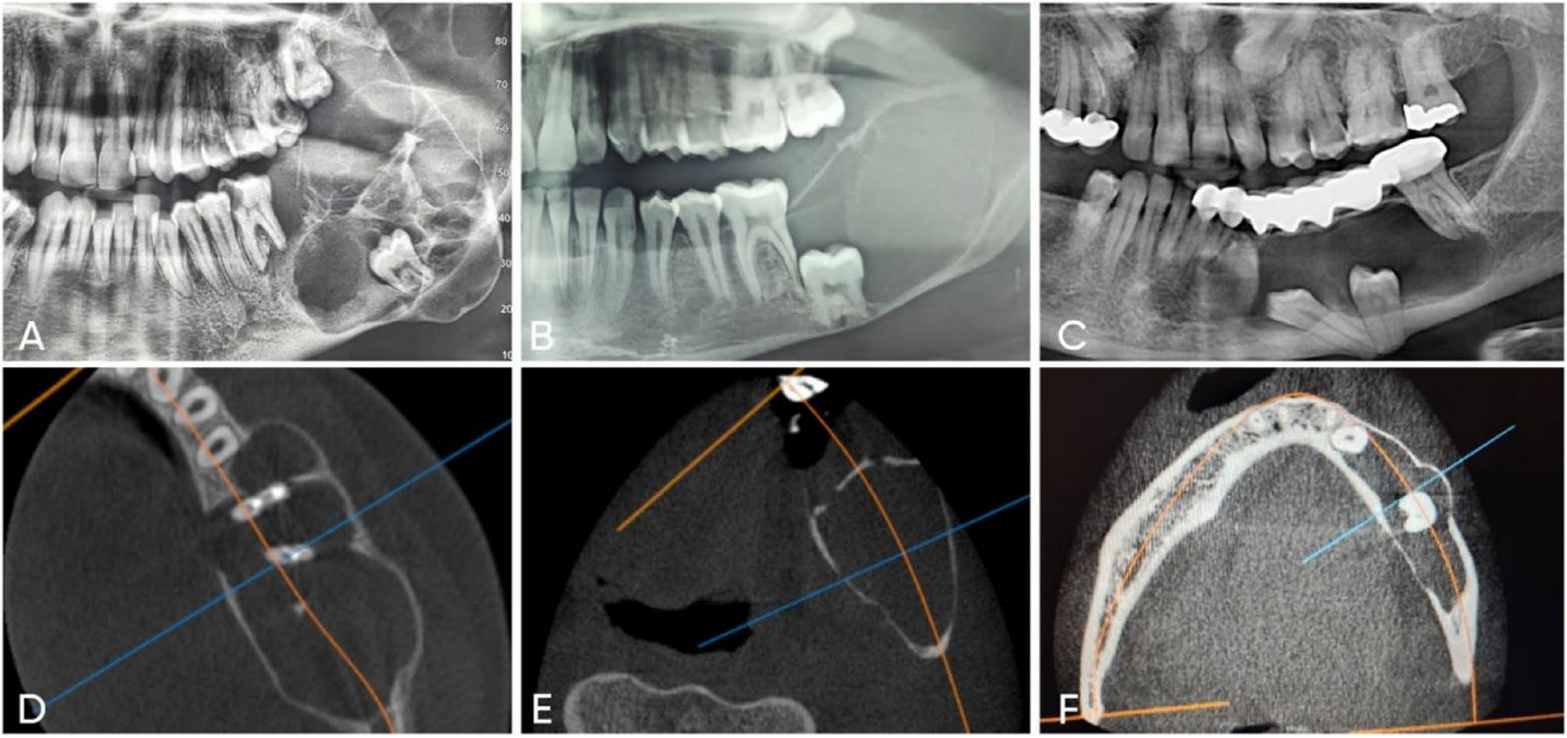
(**a**) Panoramic x-ray of ameloblastoma shows multilocular radiolucency. **b** Panoramic x-ray of odontogenic keratocyst shows the unilocular radiolucency. **c** Panoramic x-ray of dentigerous cyst shows the unilocular radiolucency. **d** Axial view of CBCT shows lingual bone perforation in ameloblastoma. **e** Axial view of CBCT shows intact buccal and lingual cortex in Odontogenic keratocyst. **f** Axial view of CBCT shows intact bone cortex in Dentigerous cyst

Ab group had a higher percentage of multilocular radiolucencies (66.7%) compared to the OKC (26.7%) and DC (13.3%) groups. OKC and DS groups had higher percentages of unilocular radiolucencies (73.3% and 86.7%) compared to the Ab (33.3%) group. The results showed a statistically significant difference in locular pattern between the groups (*p* = 0.002*) (Table 2; Fig. 1).

**Table 2:** Radiographic features

	Loculation		Bone Perforation *N* (%)	Root resorption *N* (%)
	Unilocular *N* (%)	Multilocular *N* (%)		
Ab	5 (33.3%) 10 (66.7%)		9 (60%)	8 (53.3%)
OKC	11 (73.3%) 4 (26.7%)		2 (13.3%)	1 (6.7%)
DC	13 (86.7%) 2 (13.3%)		0 (0%)	3 (20%)
Test P value	X^2^ × ^2^_=_10.08 *P* = 0.002*		_X_ X^2^_=_16.12 *P* = 0.0001*	X^2^_=_8.86 *P* = 0.003*

*Ab* Ameloblastoma, *OKC* Odontogenic keratocyst, *DC* Dentigerous cyst, *X*^2^ Chi square test

*Statistically significant (*P* ≤ 0.05)

#### Association of bone perforation

The Ab group had a higher percentage of cases associated with bone perforation (60%) compared to the OKC (13.3%) and DC groups (0%). The results revealed a significant difference in the association of bone perforation between the groups (*p* = 0.0001*) (Table 2; Fig. 1).

#### Root resorption

Eight cases of Ab, one keratocyst and 3 dentigerous cysts were associated with root resorption in one or more of included teeth. The results revealed a significant difference in the association of root resorption between the groups (*p* = 0.003*) (Table 2).

### Histopathological results

**Fig. 2:**
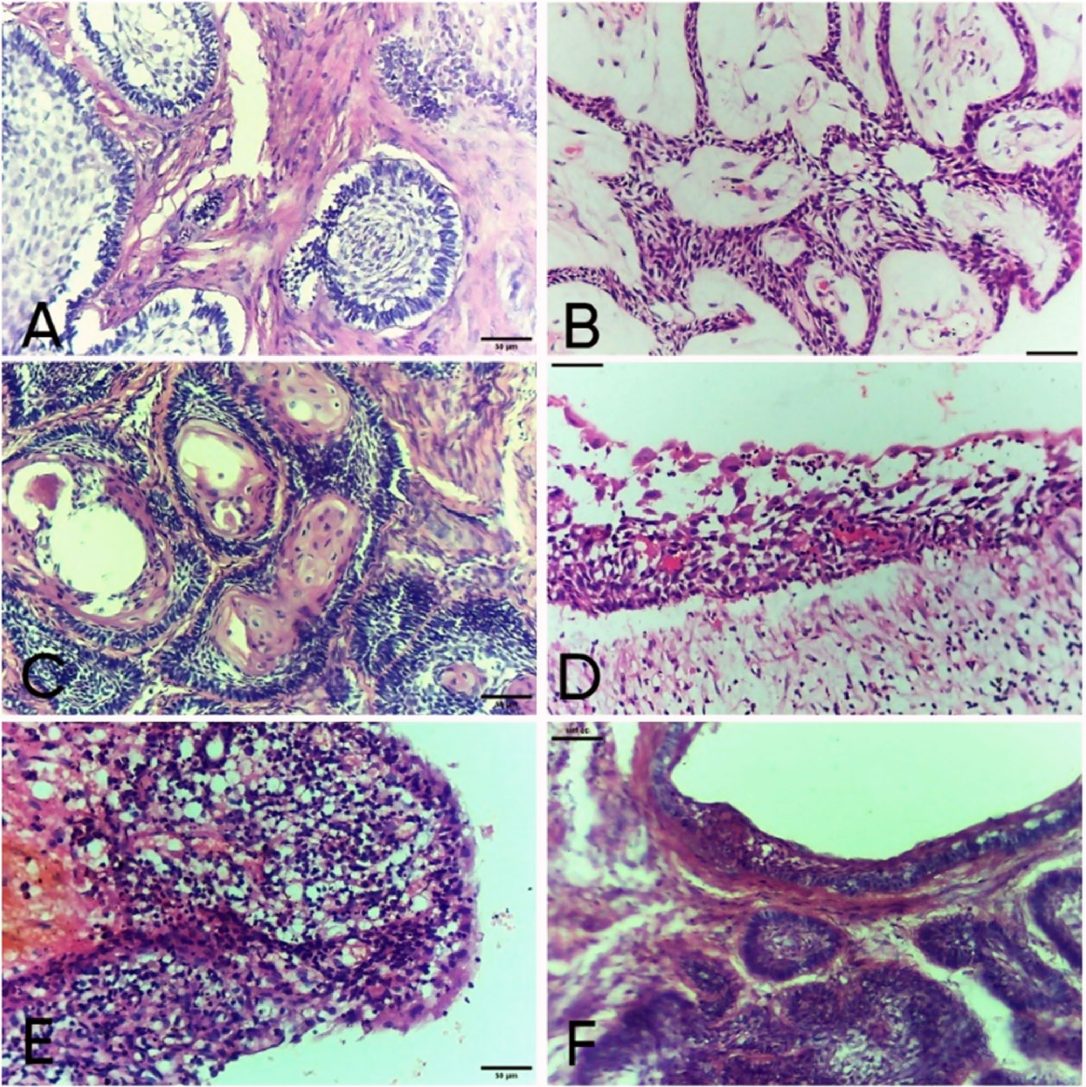
Photomicrographs of histological subtypes of conventional and unicystic ameloblastoma. (H&E ×200) Follicular ameloblastoma with odontogenic epithelial islands formed of peripheral columnal ameloblast like cells and central stellate reticulum-like cells. **b** Plexiform ameloblastoma with anastomosing strands of odontogenic epithelium with scanty stroma. **c** Acanthomatous pattern of conventional ameloblastoma revealed squamous metaplasia of stellate reticulum-like cells. **d** Unicystic ameloblastoma luminal type revealing ameloblastomatous proliferation at the level of cystic epithelial lining. **e** Unicystic ameloblastoma intraluminal type with nodule of ameloblastomatous epithelium projecting inside the cystic cavity. **f** Mural subtype showing follicles of odontogenic epithelium presented in the cyst wall of odontogenic cyst

The histopathological assessment of Ab revealed 10 cases of conventional Ameloblastoma, which were subdivided into 5 follicular, 3 plexiform, 1 acanthomatous and 1 desmoplastic variant. In addition to 5 cases of unicystic ameloblastoma subdivided into 3 mural, 1 luminal and 1 intraluminal subtypes. (Fig. 2: a-f).

Histopathological features by H&E stain for keratocyst and dentigerous cyst were shown in (Fig. 4a) and (Fig. 5a).

### Immunohistochemical results

#### SOX2 staining score

##### Ameloblastoma

Only one case showed negative immunoreaction. SOX2 gene expression was observed in fourteen cases of Ab with moderate to strong intensity. By calculating total score, 66.6% (10 cases) recorded high positive scores, 26.7% (4 cases) recorded low positive expression. (Median of total score = 4) (Table 3).

**Table 3:** SOX2 scores of staining percentage and intensity

	SOX2 staining percentage	SOX2 intensity	Total score
	score 0 score1 score2 score3 score4	score 1 score2 score 3	Negative Low High
Ab	1 4 6 4 0	3 7 4	1(6.7%) 4 (26.7%) 10 (66.6%)
OKC	0 2 8 5 0	1 3 11	0 1 (6.7%) 14 (93.3%)
DC	0 10 5 0 0	10 5 0	0 13(86.7%) 2 (13.3%)
P value	0.007*	> 0.001*	> 0.001*
P value between groups	P1 = 0.264 P2 = 0.044* P3 = 0.002*	P1 = 0.022* P2 = 0.040* P3 = < 0.001*	P1 = 0.048* P2 = 0.012* P3 = < 0.001*

Used test: Kruskal-Wallis test followed by post hoc Dunn’s multiple comparisons test

*Ab* Ameloblastoma, *OKC* Odontogenic keratocyst, *DC* Dentigerous cyst

P1: significance between AB and OKC groups, P2: significance between AB and DC groups, and

P3: significance between OKC and DC

*Statistically significant (*P* ≤ 0.05)

**Fig. 3:**
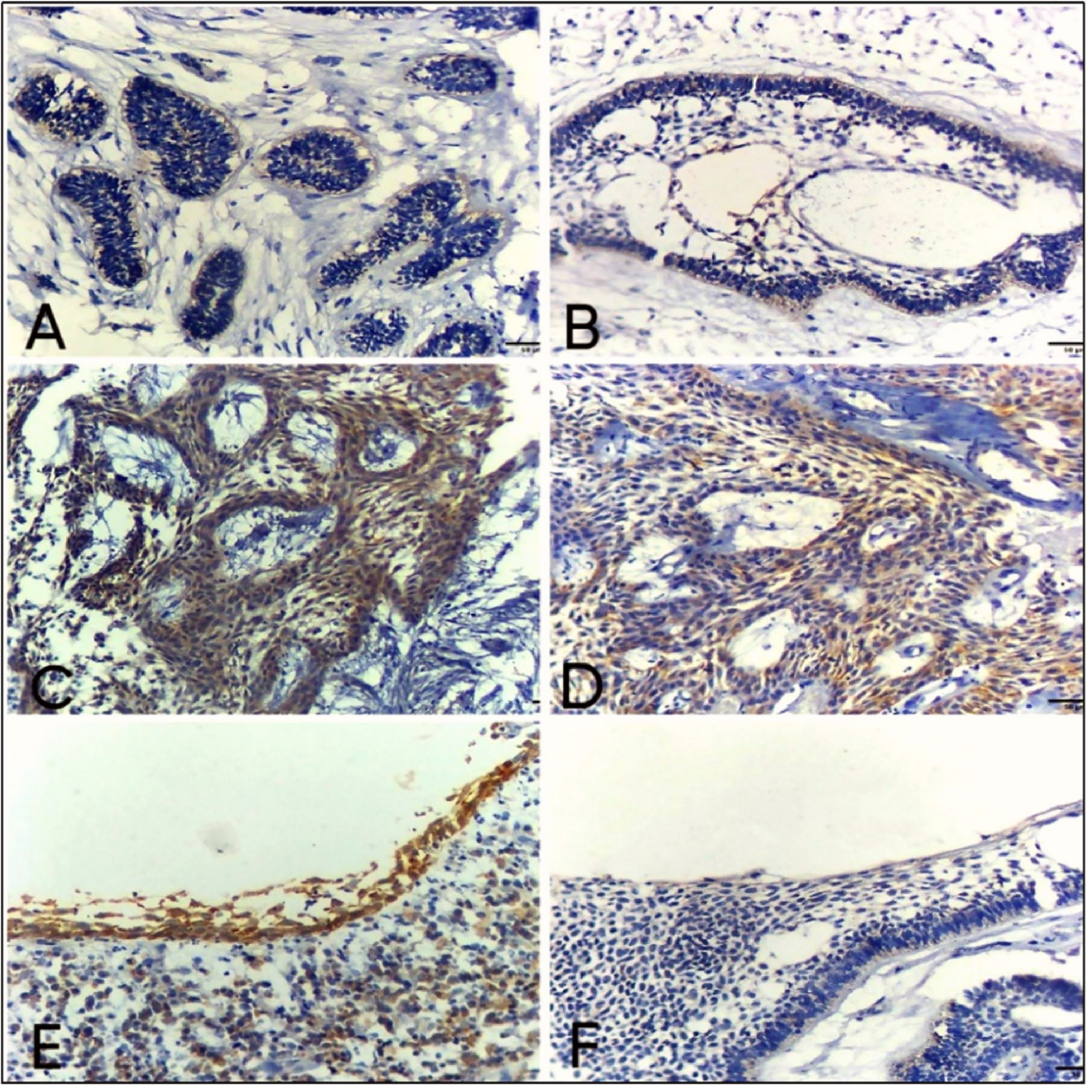
Photomicrographs of SOX2 expression in conventional and unicystic ameloblastoma. (ABC-DAB ×200) (**a**) Cytoplasmic expression of SOX2 in odontogenic epithelial follicles of follicular ameloblastoma. **b** Follicular ameloblastoma with microcystic formation with nuclear immunoreactivity for SOX2 mostly in the peripheral ameloblast like cells of odontogenic epithelial follicles. **c** Positive cytoplasmic and nuclear immunoreactivity for SOX2 in odontogenic epithelial strands of plexiform type of conventional ameloblastoma. **d** Plexiform ameloblastoma shows nuclear and cytoplasmic expression of SOX2 in ameloblast like cells and stellate reticulum-like cells in odontogenic epithelial strands. **e** Luminal unicystic ameloblastoma with positive nuclear and cytoplasmic immunoreactivity of SOX2 in proliferative ameloblastomatous epithelium and chronic inflammatory cells. (f) Mural unicystic ameloblastoma with weak positive cytoplasmic expression of SOX2 in proliferative ameloblastomatous epithelium (ABC-DAB ×200)

The expression was mainly nuclear in ameloblast like cells at the periphery of ameloblastic units, also large number of cases showed cytoplasmic expression which indicate more tumor aggressiveness. (Fig. 3a-f).

##### OKC

**Fig. 4:**
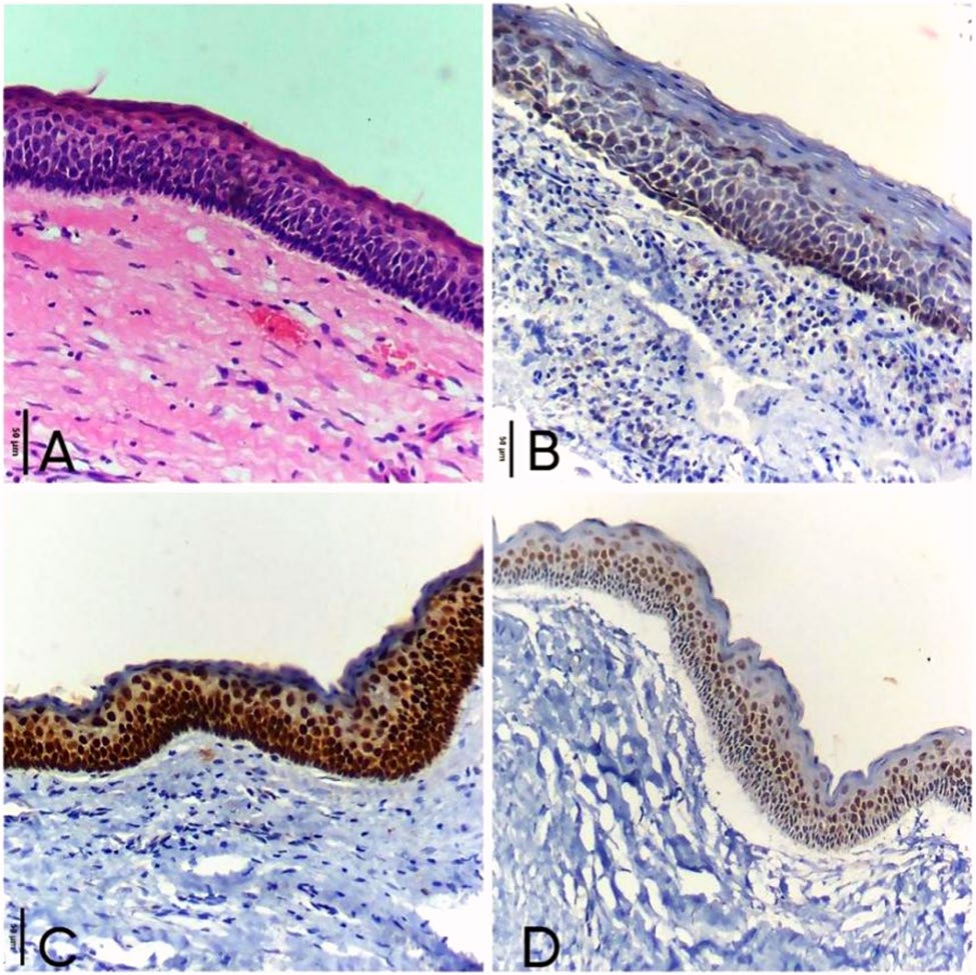
Photomicrographs show: (**a**) Odontogenic keratocyst with hyperchromatic palisaded basal cells and flat epithelial connective tissue interface (H&E ×200). **b** Moderate nuclear expression of SOX2 in basal and para-basal layers of odontogenic keratocyst epithelial lining (ABC-DAB ×200). **c** Odontogenic keratocyst with strong nuclear immunoreactivity for SOX2 in basal and para-basal cells of epithelial lining (ABC-DAB ×200). **d** Strong nuclear immunoreactivity of SOX2 in basal and para-basal cells of epithelial lining in odontogenic keratocyst (ABC-DAB ×200)

The SOX2 expression was detected in basal and supra-basal layers of all cases with high positive expression in 14 cases (93.3%) and only one case showed low positive expression (6.7%), (median of total score = 5) (Fig. 4: b-d).

##### DC

**Fig. 5:**
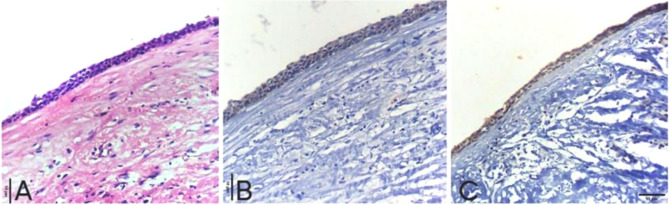
photomicrographs show (**a**) Dentigerous cyst with thin odontogenic epithelium of 2–4 cell thickness with flat epithelial connective tissue interface (H&E ×200). **b** Dentigerous cyst with weak cytoplasmic expression of SOX2 in epithelial lining (ABC-DAB ×200). **c** Moderate nuclear and cytoplasmic immunoreactivity for SOX2 in Dentigerous cyst epithelial lining (ABC-DAB ×200)

Weak to moderate intensity of SOX2 expression was observed in the cytoplasm of basal and supra-basal layers (Fig. 5b-c). By calculating total score, SOX2 expression was low positive in 13 cases (86.7%) and 2 cases recorded high positive expression (13.3%), (median of total score = 3) (Table 3).

**Table 4:** Correlation of SOX2 IHC staining scores to clinical & radiographic findings

Correlation with SOX2 IHC staining score	Spearman’s rank correlation coefficient (*r*)	*P*-value
Age	0.0242	0.874
Gender	−0.2417	0.162
Recurrence	0.467	0.001*
Root resorption	0.2807	0.062
Radiographic loculation	0.225	0.137
Bone perforation	0.307	0.040*

*Statistically significant (*P* ≤ 0.05)

Statistical analysis showed significant difference in SOX2 expression between Ab and DC (*P* = 0.012) and between OKC and DC (P = < 0.001). Additionally, OKC recorded a significantly higher SOX2 expression than Ab (*P* = 0.048).

### Correlation results

Correlation of SOX2 IHC staining scores to clinical & radiographic findings revealed a non-statistically significant correlation to age and gender, non-significant positive correlation to root resorption and multilocular radiolucency pattern. While it revealed significant medium positive correlation to bone perforation (*P* = 0.04) and a significant high positive correlation to recurrence (*P* = 0.001). (Table 4).

## Discussion

Recent studies suggest that tumor stem cells play a crucial role in the initiation, progression, aggressiveness and recurrent nature of odontogenic cysts and tumors [2, 19, 20]. This study aimed to assess the prognostic role of SOX2 as a tumor stem cell marker in odontogenic cysts and tumors; it is one of the primary studies which correlate SOX2 immunohistochemical staining scores to different clinical and radiological findings in these common pathologies.

The lesions investigated in this study were ameloblastoma and odontogenic keratocyst, as they possess great clinical interest due to their aggressive biological behavior and tendency to recurrence, and the dentigerous cyst (DC) as it is the most common developmental odontogenic cyst. The expression of stem cell marker was documented via immunohistochemistry as it is considered the gold-standard for the detection of the tissue-specific expression of proteins and their precise subcellular localization [27]. 

In this study, 15 cases of both conventional and unicystic Ab were assessed, follicular form was the most recorded pathological variant followed by plexiform. SOX2 gene expression was observed in all studied Ab cases except one case of unicystic type. These findings are in accordance with Pagella et al.,2020 [28] who reported the widespread expression of the dental epithelial stem cell marker SOX2 within Ameloblastomas, suggested that a major proportion of the cells composing these tumors present cancer stem cells-like properties and would explain its aggressive behavior.

Most of the included Ab specimens showed moderate to high SOX2 intensity. As regards SOX2 IHC total scores; 10 cases showed high positive scores and 4 cases recorded low positive scores. The expression was mainly nuclear in ameloblast like cells at the periphery of ameloblastic units, but also large number of cases showed nuclear and cytoplasmic expression. According to van Schaijik et al.,2018 [29] this cytoplasmic expression of SOX2 could be an indicator of more tumor aggressiveness. Results of this study are in accordance with Jurri et al. [30], who reported that SOX2 was expressed in the epithelial cells of follicular and plexiform ameloblastoma diffusely, suggesting that SOX2 functions in maintaining the progenitor state of epithelium in ameloblastoma.

A statistically significant higher SOX2 expression in Ab than dentigerous cyst was recorded in this study (*P* = 0.012). This result resembles the findings by Balbinot et al., 2023 [24], as they reported a greater expression of SOX-2 in Ab in relation to the DC lining epithelium and dental follicle (DF) epithelial nests.

On the other hand, de Freitas Silva et al. 2020 [31] and Bandyopadhyay et al., 2017 [32] investigations revealed that SOX2 was not expressed in Ab specimens. Phattarataratip et al., 2021 [17] recorded mild to moderate SOX2 expression, limited to the ameloblast like cells in ameloblastoma. The conflicting results in literature regarding SOX2 expression pattern in ameloblastomas are most likely due to using different antibodies. This variable SOX2 expression pattern in ameloblastomas may suggest that additional molecular mechanism could be responsible for the neoplastic characteristics of this specific odontogenic tumor.

As regards OKC cases in this study, the SOX2 expression was detected in basal and suprabasal layers of all cases with high positive expression in 14 cases and only one case showed low positive expression. This result is in accordance with Bandyopadhyay et al., 2017 [32], de Freitas Silva et al., 2020 [31] and Pereira et al., 2023 [25] in which all cases showed patent SOX2 expression in all layers of OKC.

Throughout the current analysis, the entire thickness of the OKC epithelial lining had SOX2 staining, which may suggest that all cell layers of OKC specimens retain the progenitor characteristics of odontogenic epithelium, displaying a stem cell-like phenotype and ongoing proliferation. According to de Freitas Silva et al. [31] this could suggest that OKC displays keratinocyte differentiation, and validate the epithelium’s basal and suprabasal layers’ OKC cells’ capacity for dedifferentiation. High SOX2 expression in OKC as found in our study may explain the high mitotic activity and aggressive nature of the lesion and point to the imbalance between cell growth and cell death in OKC, which could be a sign of neoplastic attitude and the unusually higher recurrence rate of OKCs than other jaw cysts. These findings provide a rationale for the reclassification of this entity as a tumor. Similarly, other studies highlighted the critical role of Sox2 in mitotic progression and stem cell maintenance [33, 34]. 

A statistically significant difference in SOX2 expression was found between OKC and Ab (*P* = 0.048), as well as DC (P = < 0.001). This was similar to de Freitas Silva et al., 2020 [31] and Pereira et al., 2023 [25] as they recorded that SOX2 immunoreactivity was statistically significant in OKC cases compared to Ab cases. Phattarataratip et al., 2021 [17] showed that most OKC (86.7%) expressed high SOX2 expression in more than 50% of epithelial cells, significantly higher than ameloblastoma. This difference may be related to their diverse cells of origin or stage of histogenesis.

In the current study, DC showed low to moderate SOX2 intensity. IHC scores were low in 13 cases of DC, while only two cases were highly positive. This result is similar to Balbinot et al., 2023 [24] where all 10 cases of DC showed low positive expression. Furthermore, Phattarataratip et al., 2021 [17] recorded minimal to no SOX2 expression in DC cases. This proves the fact that DC is not aggressive in its biological behavior, as it is generally accepted that DC is developed from the accumulation of fluid between the reduced enamel epithelium and the crown of an unerupted tooth, while OKC is believed to be derived from the remnants of dental lamina.

 As regards SOX2 staining pattern, Sox2 is classically described as a nuclear transcription factor; however, its localization is not exclusively nuclear. Several recent studies, consistent with our findings, also reported both cytoplasmic and nuclear immunoreactions for SOX2, with intense staining in malignant and aggressive odontogenic tumors [35, 36]. Mannava C et al.,2025 [37] reported that SOX2 expression was observed in odontogenic keratocysts with diffuse, nuclear, and cytoplasmic positivity across the full epithelial thickness. Cytoplasmic localization has been interpreted as evidence of Sox2 protein retention, post-translational modification, or a mechanism of regulating its nuclear availability [36–38]. Schaefer T et al., 2024 [38] proposed a revised model of SOX2 action confirming that SOX2 modulates translation independent of nuclear structures, It concludes that nuclear and cytosolic fractions cooperate to impose cell fate decisions via both transcriptional and translational mechanisms.

This indicates that while nuclear SOX2 drives stemness and pluripotency, its presence in the cytoplasm may have distinct, and as yet not fully understood, functions in odontogenic tumor progression. This may reflect the biological behavior of odontogenic tumors, in which Sox2-positive cells exhibit altered trafficking or retention within the cytoplasm, potentially influencing their stemness-related functions.

 Regarding clinical correlations, the mean age of included patients in Ab, OKC & DC groups was 33.80 ± 14.65, 32.67 ± 15.01, and 28.07 ± 10.87 respectively. This was in accordance with the globally reported peak of incidence as reported in many studies [39–41]. Statistically, there was non-significant correlation between IHC scores and patient age (*P* = 0.874). Concerning gender domination, the results of this study displayed that males are more affected than females in all studied groups; Ab, OKC and DC as the percentage of male patients was 66.7%, 60% and 53.3% respectively. This result seems in line with other studies [42–46], but in contrast with some results in literature [47] which showed no sex predilection in Ab, OKC and DC. Correlation was not statistically significant between IHC scores and patient gender (*P* = 0.162).

In the current work, the most affected site in Ab, OKC and DC was posterior mandibular area with percentage 66.7%, 80% and 66.7% respectively. This result is compatible with most of the reported results in the literature [48–50]. This predominance of posterior mandible may be due to its abundant blood supply, which provides tumors with the oxygen and nutrients they need for survival [51]. 

Five cases were received as recurrent in our study; 3 recurrent cases were Ab, representing 20% of the group. This result is in line with Lei et al., 2024 [18] and Bwambale et al., 2021 [39] studies, in which the recurrence rate was 17.2% and 23.2% respectively. However, Yang et al., 2017 [52] reported a recurrence rates of 9.8%. In association with histopathological patterns, the recurrent cases in this study were more in follicular variant which agrees with Goh et al., 2021 [44] and Hresko et al., 2022 [53]. In contrast, Bwambale et al., 2021 [39] reported that plexiform pattern had a higher recurrence rate. According to Au et al., 2019 [54], there is no correlation between histological patterns and ameloblastoma recurrence.

Among OKC group, 2 cases were received as recurrent with 13.3% percentage. This percentage is similar to Rahman et al., 2019 [55]. High recurrence rate may be due to the presence of satellite cysts and the thin cystic epithelium, which has a lower tensile strength than other maxillofacial tumors, making it challenging to remove the tumor entirely in toto [56, 57]. Additionally, dedifferentiation of the basal and suprabasal cells in OKC may be the cause of satellite cyst-induced recurrence [25, 32]. 

A significant positive correlation was found between IHC scores and recurrence (*P* = 0.001) as the specimens of recurrent cases showed strong SOX2 overexpression. Similarly, Tseng et al., 2022 [58], found that recurrent cases of Ab showed much higher labeling indices of SOX2 than paired primary lesions, and proposed that recurrence of Ab is associated with the presence of SOX2-expressing stem cells, as some tumor nests might be remained when the tumor was not adequately removed, SOX2 expressing cells remaining in these nests might give rise to the recurrent disease. Vanner et al. [59] showed that the SOX2 cells were quiescent in comparison to other tumor cells that cycled quickly, and that these SOX2 cells cause tumor regrowth following therapy. These findings suggest that en block resection is still the treatment of choice for locally aggressive odontogenic lesions. Targeted therapies to specifically target SOX2-expressing stem cells may be developed for more effective treatment.

 Regarding radiographic results, 66.67% of the Ab showed multilocular radiolucency, these results were comparable to the radiographic image reported in Smit et al., 2024 [40] and Malik et al., 2018. [60] Boffano et al., 2021 [61] mentioned that multilocular radiographic appearance of Ab was associated with high risk of recurrence. In this study, 73.3% of OKC patients and 86.7% of DC cases had clearly defined unilocular radiolucency, which is consistent with findings of Rahman et al. 2019 [55], and Terauchi et al., 2019. [62] However some big cysts exhibit a multilocular picture on panoramic radiographs, which is believed to be caused by the persistence of bone trabeculae within the radiolucency [63]. 

No significant correlation was found between SOX2 expression and the radiographic locular pattern of lesions in this study (*P* = 0.137), as well as root resorption of included teeth (*P* = 0.062).

Current study showed cortical bone perforation in 60% of Ab cases in computed tomography x-ray (CBCT) which agrees with Smit et al., 2024 [40] who recorded cortical perforation in 77% of cases, this high percent points to the aggressiveness of this lesion. Two cases showed bone perforation in OKC, which demarcated the growth behavior of these lesions as the OKCs develop Antero-posteriorly with little initial sign of cortical expansion [64]. However, other studies reported higher incidence of bone perforation associated with keratocyst (71.4%) [65]. 

A significant correlation was found between SOX2 and cortical bone perforation (*P* = 0.04). This result is in accordance with Carneiro et al. [65], who correlated the imaging aspects of odontogenic cysts and tumors in CBCT to the lesion behavior, and reported that cortical perforation is the most indicative factor of an aggressive behavior. His results similarly to ours showed that aggressive lesions including ameloblastoma and keratocyst were highly associated with cortical bone perforation while among the non-aggressive lesions, the dentigerous cyst was not associated with cortical perforation. Fidele et al. [66] reported that enucleation of keratocysts combined with cortical perforation was statistically associated with high recurrence rate.

The results suggest notable link between Sox2 expression and indicators of bone resorption, including bone perforation and root resorption. Over expression of Sox2 in lesions showing bone perforation supports its role in modulating osteolytic activity. This is in accordance with other studies demonstrate that overexpression of SOX2 enhances osteoclast formation and bone-resorbing activity [67] and also reduces the osteogenic differentiation potential of stem cells [68]. This correlation supports the potential prognostic value of Sox2 as a marker of aggressiveness in odontogenic lesions.

The association between SOX2 expression and cortical bone perforation likely reflects not only stemness but also involvement in tumor-driven bone homeostasis. Recent studies indicate that SOX2 is involved in modulating signaling pathways relevant to bone homeostasis and osteoclast differentiation. Shen et al., 2020 [67] demonstrated that SOX2 overexpression-induced increase in osteoclast differentiation via EGFR/ERK and NFATc1 pathways. Seo E et al. [69] reported that SOX2 maintains osteoprogenitor self-renewal and delays differentiation, which may shift the balance toward resorption. Additionally, in experimental periodontitis by Gu & Ding, 2023 [70], the miRNA-200c/SOX2 axis has been shown to modulate alveolar bone resorption via RANKL changes. These studies support the possibility that SOX2-positive odontogenic tumor cells may contribute to local bone resorption and cortical perforation through similar molecular mechanisms.

All these findings which observed a strong positive expression of SOX2 transcription factor in OKC and Ab suggest that these lesions have cells with characteristics of cancer stem cells that could be related to the progression and recurrence of these aggressive odontogenic pathologies. Developing targeted therapies for these stem cell populations could offer significant clinical benefits by enabling more effective and less aggressive treatment strategies.

To sum up, this study showed that SOX2 elevated expression in odontogenic lesions with higher recurrence and invasive behavior underscores its potential utility in predicting clinical outcomes and poor prognosis. Moreover, the association of Sox2 expression with clinical features such as bone perforation and recurrence implies its involvement in the local aggressive behavior of these lesions. These findings support the potential prognostic significance of Sox2 as a marker of aggressiveness in odontogenic lesions, particularly in identifying lesions with a higher risk of recurrence. Sox2 immunostaining could thus act as a prognostic tool to aid in stratifying patients for closer follow-up or specific surgical intervention to lower patient morbidity and improve the treatment outcome.

Future studies should integrate bioinformatics and molecular biology approaches elucidate the SOX2-mediated pathways at the gene levels. Techniques such as RNA sequencing or microarray analysis could provide a comprehensive profile of genes differentially regulated by SOX2 in aggressive lesions. Additionally, functional assays such as SOX2 knockdown/overexpression in odontogenic tumor cell lines, coupled with pathway analysis, could validate SOX2 role in recurrence and invasiveness. These approaches may also uncover potential therapeutic targets for precision treatment.

### Limitations and further studies

Despite its valuable insights, the study has some limitations. The sample size was relatively small, particularly recurrent cases, the lack of longitudinal follow-up data restricted our capacity to assess the prognostic value of Sox2 in predicting actual recurrence of odontogenic lesions. Furthermore, variations in the treatment modalities received by patients were not fully accounted for, which could potentially influence recurrence patterns independently of Sox2 expression. Future studies with larger cohorts, incorporating other molecular techniques, functional analyses and long-term follow-up are warranted to validate Sox2 as a prognostic biomarker in odontogenic tumors and cysts.

## Conclusion

In conclusion, this study provides evidence that Sox2 is significantly over-expressed in ameloblastoma and odontogenic keratocyst (OKC) compared to dentigerous cyst (DC), reflecting its association with lesions known for greater local aggressiveness and higher recurrence potential. Given its established function as a tumor stem cell marker, Sox2 likely contributes to the maintenance of a self-renewing, proliferative cell population within these lesions.

The positive correlation between Sox2 expression and aggressive features such as bone perforation and recurrent lesions further underscores its role in promoting invasive behavior. These findings support the prognostic value of Sox2, as its elevated expression may serve as a biomarker for identifying odontogenic lesions with a higher risk of recurrence and aggressive clinical behavior. This could be a valuable tool in improving patient management.

Within the limitations of this study, the differential expression pattern of Sox2 provides a promising foundation for its future use as a prognostic indicator. Continued investigation with larger cohorts and follow-up data is warranted to validate the clinical utility of Sox2 as a tumor stem cell–associated prognostic marker in odontogenic tumors and cysts.

## Data Availability

The corresponding author can provide the data sets utilized and/or analyzed for this study upon reasonable request **.**

## Abbreviations

TSCs: Tumor stem cells
SOX-2: Sex-determining region Y (SRY)-box 2
Ab: Ameloblastomas
OKC: Odontogenic Keratocyst
DC: Dentigerous cyst
IHC: Immunohistochemical
OTs: Odontogenic tumors
OPG: Panoramic X-ray
CSCs: Cancer stem cells
IRB: The Institutional Review Board
PBS: Phosphate buffered solution
DAB: Diaminobenzidine tetrahydrochloride
SCC: Squamos cell carcinoma
DF: Dental follicle
(EGFR): Epidermal growth factor receptor
(ERK): Extracellular signal-regulated kinase (NFATc1):nuclear factor of activated T-cell c1
RANKL: Receptor activator of nuclear factor κB ligand
